# Establishment of a combination scoring method for diagnosis of ocular adnexal lymphoproliferative disease

**DOI:** 10.1371/journal.pone.0160175

**Published:** 2017-05-16

**Authors:** Xiao-Li Qu, Yan Hei, Li Kang, Xin-Ji Yang, Yi Wang, Xiao-Zhong Lu, Li-Hua Xiao, Guang Yang

**Affiliations:** 1 Ophthalmology Department, Qianfoshan Hospital, Shandong Province, China; 2 Institute of Orbital Disease, General Hospital of Chinese People’s Armed Police Forces, Beijing, China; 3 Beijing Institute of Basic Medical Sciences, Beijing, China; Monash University, AUSTRALIA

## Abstract

Lymphoproliferative diseases (LPDs) of the ocular adnexa encompass the majority of orbital diseases and include reactive follicular hyperplasia (RFH), atypical lymphoid hyperplasia (ALH), and mucosa-associated lymphoid tissue lymphoma (MALToma). Lymphoid follicles (LFs) are usually observed during the histological examination of LPDs. Currently, because there is a lack of specific clinical signs and diagnostic immunohistochemical biomarkers, it is difficult for pathologists to distinguish MALToma from ocular RFH and ALH, which makes the clinical management of these conditions difficult. Here, we analyzed the clinical features of patients with ocular adnexal LPDs (*n* = 125) and investigated the structure of LFs in paraffin-embedded tissue samples using anti-CD23 and anti-IgD immunochemistry. We found that some clinical features including age, sex, and laterality were different among RFH, LFH, and MALToma. Additionally, immunohistochemistry revealed that the expression of IgD and CD23 was higher in RFH patients and decreased in patients with ALH and MALToma. Moreover, LFs in RFH were intact, whereas the structures of most LFs were disrupted in ALH. In MALToma specimens, few intact LFs were observed. In a further investigation, we combined the results for CD23/IgD immunohistochemistry and the structure of LFs to establish a scoring method for the differential diagnosis of LPDs. According to the BIOMED-2 protocol, we further detected IgH gene monoclonal rearrangement in 73 cases (35 RFH, 17 ALH, and 21 MALToma cases). The sensitivity of our scoring method, based on a comparison with the results of IgH gene monoclonal rearrangement detection, was 85.7% (18/21) for MALToma and 35.3% (6/17) for ALH. Our study provides a method that may be useful for the differential diagnosis of RFH, ALH, and MALToma.

## Introduction

Lymphoproliferative disease (LPD) of the ocular adnexa is a relatively common orbital disease and is reported to account for 10.0–24.7% of primary ocular adnexal tumors [1,2]. Ocular adnexal LPDs can arise within the intraconal and extraconal orbit soft tissues, lacrimal gland, extraocular muscles, lacrimal sac, eyelids, or conjunctiva. Ocular adnexal LPD is a heterogeneous group that is mainly divided into three subtypes: reactive follicular hyperplasia (RFH), atypical lymphoid hyperplasia (ALH), and extranodal marginal zone lymphoma of mucosa associated lymphoid tissue lymphoma (MALToma). Most of these primary tumors are B-cell-derived.

MALToma has been recognized as the most common histologic type of ocular adnexal lymphomas and is reported to comprise approximately 35–90% of primary ocular adnexal lymphomas [2–5]. Classical MALToma is a low-grade, extranodal, marginal zone non-Hodgkin’s B-cell lymphoma. RFH is considered to be a benign and reversible hyperproliferative condition that presents as a mass-like lesion characterized by many LFs with infiltration of mature plasma cells and histiocytes [6]. In contrast, ALH is an equivocal lymphoproliferative lesion that cannot be diagnosed as definitely benign or malignant and is characterized by serious clinicopathologic features that are still insufficient to justify a malignant diagnosis [7,8]. It has been suggested that ocular adnexal LPDs might arise from chronic inflammatory or autoimmune disorders [3,9–14].

It is nearly impossible to categorize all ocular LPDs into clearly defined types, since the histological features might vary extensively among different cases. For instance, lesions often share certain overlapping morphological features, particularly when the tissue becomes infiltrated with clusters of small B-cells with scattered centrocyte/centroblast-like, plasmacytoid, and monocytoid cells or LFs. Thus, the histopathological diagnosis of RFH, ALH, and MALToma in the ocular adnexa is relatively difficult for clinicians and pathologists, which makes choosing a therapeutic method difficult. Currently, molecular genetic analysis and immunophenotyping techniques are commonly applied to assist pathologists in obtaining an accurate diagnosis [1,15,16], but the feasibility and consistency of these procedures are still not adequate in most hospitals.

The secondary LFs in peripheral lymphoid organs are key sites for somatic hypermutation and immunoglobulin (Ig) class switching to generate mature B-cell populations for the humoral immune response [17–20]. LF formation depends on the activation of naïve B-cells by T-cells capable of recognizing epitopes of the same antigenic complex [19,21]. In peripheral lymphoid tissues, transmembrane immunoglobulin D (IgD) is strictly expressed by naïve mature B-cells that form a mantle zone (MZ) as an approximately smooth curved contour of the LF, whereas CD23^+^ follicular dendritic cells (FDCs) exist in the germinal center (GC) as a high-density meshwork encompassed by the MZ [22, 23]. Thus, a typical secondary LF is marked by an IgD^+^ MZ and an IgD^−^CD23^+^ GC. LFs can be observed in all three subtypes of LPDs within the ocular adnexa; however, their presence varies among diseases. Therefore, we sought to determine whether the differential diagnosis of ocular adnexal RFH, ALH, and MALToma could be performed based on CD23/IgD immunostaining and clinicopathological manifestations. We also analyzed the associations of CD23 and IgD among the three LPD subtypes in a Chinese population.

## Materials and methods

### Study subjects

We performed a retrospective study of 125 patients with primary ocular adnexal LPD who were diagnosed at the Institute of Orbital Disease, General Hospital of Chinese People’s Armed Police Forces (Beijing, China) from January 2006 to September 2014. The study population was divided into three subgroups based on diagnosis: RFH (*n* = 54), ALH (*n* = 28), and MALToma (*n* = 43). Every case was reviewed by two experienced pathologists to reach a consistent diagnosis according to the criteria of the 2008 World Health Organization (WHO) Classification of Tumor of Hematopoietic and Lymphoid Tissues [23]. Patients with inadequate clinical information or insufficient paraffin-embedded tumor specimens were excluded from this study. Demographic profiles (age, sex), clinical information (primary presentation, signs, symptoms, medical treatment history, and evidence of systemic disease) and histological diagnostic records (sample site and immunohistochemical manifestation) were extracted from surgical pathology reports. Computed tomography and/or magnetic resonance imaging scans, if available, were reviewed to localize the site and extent of disease. Clinical staging was performed for all MALToma patients according to the Ann Arbor classification. All primary MALToma patients were in stage IE. After surgical resection under general anesthesia, MALToma patients were sent to an oncologist for further treatment. All patients were sero-negative for human immunodeficiency virus and none of them had severe systemic diseases or other malignant tumors. The research protocol was reviewed and approved by the ethics committees and institutional review board of the General Hospital of Chinese People’s Armed Police Forces, and the study was conducted according to the principles of the Declaration of Helsinki. Written informed consent for the collection of medical information and specimens was obtained from patients or their guardians at the first visit.

### Histopathology and immunohistochemistry

Tumor specimens were fixed in 10% (v/v) neutral buffered formalin and embedded in paraffin. The fixation time depended on the size of the tissue block and the tissue type. Serial sections (2 μm) were cut using a microtome and affixed onto positively charged slides. All slides were incubated at 60°C for a few hours to allow the sections to adhere to the slides. Then, tissues were deparaffinized and rehydrated through graded xylene and alcohol. Hematoxylin–eosin and immunohistochemical (IHC) staining procedures were performed according to routine protocols. Heat-induced epitope retrieval was performed using a pressure cooker in ethylenediaminetetraacetic acid buffer at pH 9.0. Primary antibody incubations were performed according to the manufacturer’s instructions. Antibodies were purchased from the following sources: anti-CD3 (ZA-0503), anti-CD20 (ZA-0549), anti-CD21 (ZA-0525), anti-CD23 (ZA-0516), and anti-Ki-67 (ZA-0502) were from ZSGB-BIO (Beijing, China) and anti-IgD (ab124795) was from Abcam (Cambridge, UK). All images were acquired on a DM2500 microscope (Leica, Wetzlar, Germany).

Analysis of IgD and CD23 immunostaining was performed by two independent histopathologists. Tonsil specimens from adenoid hypertrophy patients and samples processed without primary antibody were used as positive and negative controls, respectively. To control for inter-observer variation, IHC was scored twice at different institutions in a blinded manner. Five different high-power fields (HPF, ×400) for each section were counted, and the average number of positive cells per HPF was calculated. The variation between the two scorers was < 5%.

According to previous studies on the FDC meshwork and MZ in small B-cell lymphomas [24–26], we classified the characteristics of LF and CD23/IgD staining into four categories for each parameter and scored them as described in Tables 1 and 2.

**Table 1:** Immunostaining scoring criteria for CD23/IgD expression level.

Expression score	CD23/IgD expression level (%)
**0**	0
**1**	1–10
**2**	11–20
**3**	≥ 21

**Table 2:** Immunostaining scoring criteria for lymphoid follicle structure.

LF score	Lymphoid follicle structure
**0**	No staining or few scattered positively stained cells
**1**	Some scattered disrupted fragments with no intact lymphoid follicles
**2**	Some intact lymphoid follicles and scattered FDC/MZ fragments
**3**	Most lymphoid follicles are intact, accompanied by some disrupted fragments

### Molecular analysis of IgH gene rearrangements

Genomic DNA was extracted from formalin fixed paraffin embedded tissue using a QIAGEN DNA extraction kit. Extracted DNA was kept at −20°C. PCR amplifications of IgH rearrangements were performed using consensus primers (FR1, FR2, and FR3) based on the strategy of BIOMED-2 [20]. The PCR reaction mixture and the cycling conditions also followed the BIOMED-2 protocol. PCR products were analyzed by electrophoresis in a 2% (w/v) agarose gel containing GoldView™. We performed denaturation and renaturation of PCR products to distinguish between clonal and diverse products following the methods of Langerak et al [27]. Each PCR reaction was performed with a negative control (without genomic DNA) and a positive control (testified IgH rearrangement sample). Clonal rearrangements were assigned as one or two identical band(s) in the appropriate size range. Polyclonal patterns were characterized by the presence of a smear or multiple bands in the gels.

### Statistical analysis

The data were statistically analyzed using SPSS for Windows (Version 17.0, SPSS Inc., Chicago, IL, USA). Results were expressed as the means ± standard deviation. Differences in surface biomarker expression among groups were analyzed using unpaired Student’s *t* tests. Differences in clinical characteristics among subgroups were determined by χ^2^, two-tailed Fisher’s exact, least significant difference analysis of variance, or Student’s *t* tests, as appropriate. Differences of *P* < 0.05 or *P* < 0.05/*n* (correct level for multiple comparisons, *n* = number of multiple comparisons) were considered statistically significant.

## Results

### Clinical characteristics of the patient population

The clinical characteristics of the patient population are summarized in Table 3. Notably, several trends towards differences in the clinical characteristics could be distinguished among the three disease types. There was a large variation in patient age (13–93 years), with mean ages of 55.92, 60.44, and 64.95 years (ranges: 13–82, 23–77, and 35–93 years) in the RFH, ALH, and MALToma groups, respectively. Patient age also tended to increase with LPD progression. The age of patients with RFH was significantly lower than that of patients in the MALToma group (*P* < 0.001). Moreover, 28/54 RFH patients (51.85%) were female, whereas 9/43 MALToma patients (20.93%) were female (*P* = 0.002). The time from symptom onset to final diagnosis varied from 0.5 to 360 months in all patients. The mean disease duration for RFH was 50.38 months, which seemed longer than that for ALH and MALToma, although the difference was not statistically significant (*P*_RFH & ALH_ = 0.027, *P*_RFH & MALT_ = 0.025).

**Table 3:** Clinical features of 125 patients with ocular adnexal LPDs.

Clinical parameters	RFH (%)(*n* = 54)	ALH (%)(*n* = 28)	MALToma (%)(*n* = 43)	*P*	*P*_RFH&ALH_	*P*_RFH&MALToma_	*P*_ALH&MALToma_
**Age (years)**				0.002	0.199	< 0.001	0.065
Mean ± standard deviation	55.92 ± 12.85	60.44 ± 12.99	64.95 ± 12.16				
Range	13–82	23–77	35–93				
**Sex**				0.006	0.089	0.002	0.289
Female	28 (51.85)	9 (32.14)	9 (20.93)				
Male	26 (48.15)	19 (67.86)	34 (79.07)				
**Duration until diagnosis (months)**	0.5–360	1–120	1–240	0.028	0.027	0.025	0.812
**Anatomical site**							
Orbital soft tissue	32 (59.26)	25 (89.29)	41 (95.35)	< 0.001	0.005	< 0.001	0.376
Conjunctiva	0 (0)	2 (7.14)	8 (18.60)	0.004	0.114	0.001	0.296
Lachrymal gland	31 (57.41)	8 (28.57)	6 (13.95)	< 0.001	0.013	< 0.001	0.130
Extraocular muscle	14 (25.93)	11 (39.29)	12 (27.91)	0.434	0.213	0.827	0.317
Other sites*	5 (9.26)	2 (7.14)	2 (4.64)	0.166	0.06	0.326	0.327
**Laterality**				0.001	0.015	0.001	0.732
Unilateral	32 (59.26)	24 (85.71)	38 (8 8.37)				
Bilateral	22 (40.74)	4 (14.29)	5 (11.63)				
**Clinical symptoms**							
Periorbital swelling	40 (70.07)	22 (78.57)	35 (81.40)	0.685	0.653	0.392	0.770
Proptosis	46 (85.19)	22 (78.57)	37 (86.05)	0.683	0.593	0.905	0.521
Impaired vision	3 (5.56)	1 (3.57)	8 (18.60)	0.051	1.00	0.057	0.078
Epiphora	2 (3.70)	1 (3.57)	4 (9.30)	0.449	1.00	0.401	0.642
Pain	1 (1.85)	1 (3.57)	1 (2.33)	0.896	1.00	1.00	1.00
Motility impairment	4 (7.41)	0 (0)	10 (23.26)	0.002	0.294	0.041	0.005
Ptosis	2 (3.70)	1 (3.57)	5 (11.63)	0.245	1.00	0.236	0.392

* “Other sites” includes the eyelid, nasolacrimal duct, and nerve.

In our analysis, the majority of LPDs occurred in the orbit soft tissue, conjunctiva, lachrymal gland, and extraocular muscle. Some cases involved the eyelid, nasolacrimal duct, and nerves of the orbit. Tumors were found in two or more anatomical compartments in 59/125 (47.2%) patients. The orbit soft tissue was the most frequently involved location among all cases (98/125, 78.4%), and was involved at a particularly high frequency in MALToma patients (41/43, 95.35%), whereas lachrymal gland involvement was more prevalent in RFH patients than in the other two groups (31/44, 57.4%; *P* < 0.0167). In contrast, conjunctiva involvement was rarely present in RFH and was more prevalent in ALH and MALToma (*P* < 0.0167). Furthermore, 22/54 (40.7%) of RFH cases presented with bilateral lesions, whereas unilateral involvement was more common in ALH and MALToma.

Periorbital swelling and proptosis were the most common clinical symptoms in all patients (77.6% and 84.0%, respectively). Other symptoms, such as vision and motility impairments, pain, ptosis, and epiphora, were also frequently present. No ALH patients presented with motility impairment, which occurred in 23.26% of MALToma cases. No significant differences were observed in other clinical symptoms.

### IgD and CD23 expression in ocular adnexal LPDs

**Fig 1:**
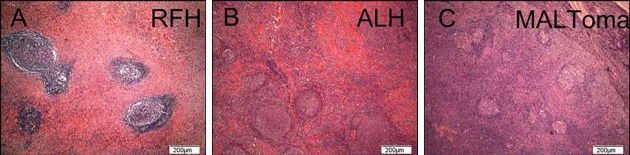
Hematoxylin–eosin staining of RFH, ALH, and MALToma. (A) RFH, (B) ALH, (C) MALToma. Various follicle-like structures accompanied by lymphocyte infiltration were observed in these three diseases by hematoxylin–eosin staining. Lymphocytes in the interfollicular areas showed no atypia.

**Fig 2:**
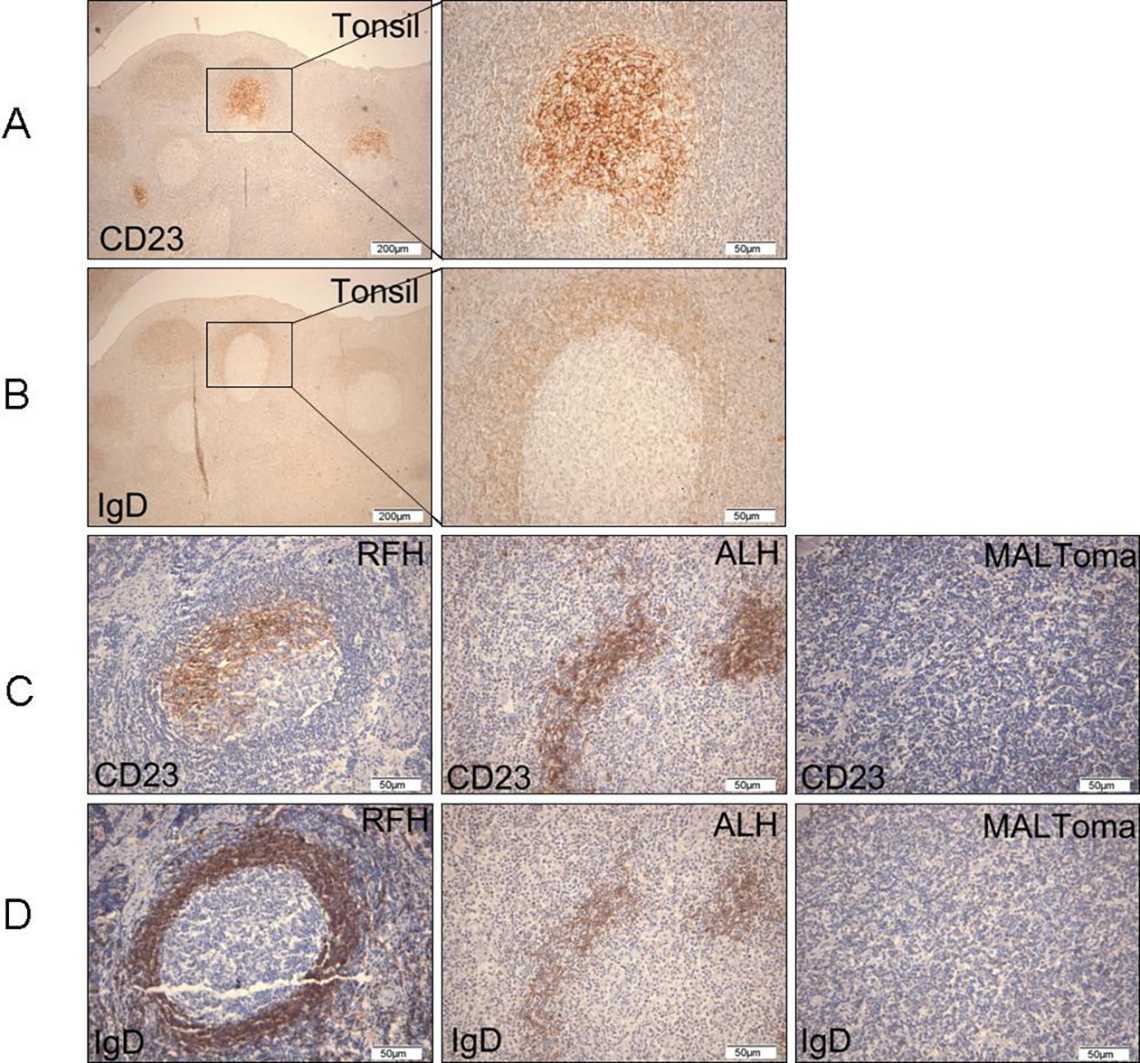
Staining patterns of IgD and CD23 in tonsil, RFH, ALH, and MALToma tissues. (A and B) Tonsil specimens showed typical secondary follicles containing an IgD^−^CD23^+^ half-moon-shaped FDC network in the GC and a surrounding IgD^+^ oval- to round-shaped mantle circle. The high-magnification composite figures show the immunoarchitectural pattern of a follicle nodule. (C and D) Follicles in RFH were characterized by prominent CD23^+^ GCs and the preservation of IgD^+^ MZs. The FDC meshwork and MZ fragments were irregularly shaped and scattered in ALH tumors. The FDC CD23^+^ meshwork was broken into clusters. Few CD23^+^ FDC meshwork fragments and IgD^+^ B-cells were observed in MALToma.

As the three types of ocular adnexal LPDs have different clinical features, we compared the pathological features of tumors from patients in the three groups. Hematoxylin–eosin staining revealed LF-like structures in the tissues of the three disease groups (Fig 1). Given that typical LFs contain IgD^+^ follicular MZ and IgD^−^CD23^+^ GC, we detected the LFs using IHC in a further analysis. First, we detected the expression of CD23 and IgD in tonsil specimens, because tonsil is a secondary lymphoid organ that contains normal LFs. The paracortical area of the tonsil specimens contained typical intact GCs and MZs, which were accompanied by variable numbers of plasma cells and histiocytes. The FCD network in GCs showed a strong immunoreactivity to the monoclonal antibody against CD23 and contained a half-moon-shaped IgD^−^CD23^+^ net (Fig 2A). The MZ surrounding the GC appeared as an IgD^+^ oval- or round-shaped circle comprised of small- to medium-sized lymphocytes with round or slightly indented nuclei (Fig 2B). We further assessed the presence of LFs in the RFH, ALH, and MALToma specimens. Variations in LF size and the frequencies of prominent CD23^+^ GCs and intact IgD^+^ MZs were observed in tissues from RFH patients (Fig 2C and Fig 2D). Additionally, a CD23^+^ half-moon-shaped FDC meshwork was apparent at the follicle light zone. Conversely, the ALH specimens showed scattered, residual reactive LFs and destroyed GCs. The FDC structure in ALH was dispersed. Most FDC networks in ALH specimens displayed irregularly shaped, disrupted, or fragmented clusters of FDC networks with scattered, disrupted IgD^+^ MZ fragments (Fig 2C and Fig 2D). IHC of MALToma samples revealed the presence of very few LFs. Moreover, MALToma samples showed a prominent lack of both a CD23^+^ FDC meshwork and IgD^+^ mantle fragments, and few CD23^+^ or IgD^+^ lymphocytes were observed (Fig 2C and Fig 2D).

### Establishment of a combination scoring method for the diagnosis of LPDs

**Fig 3:**
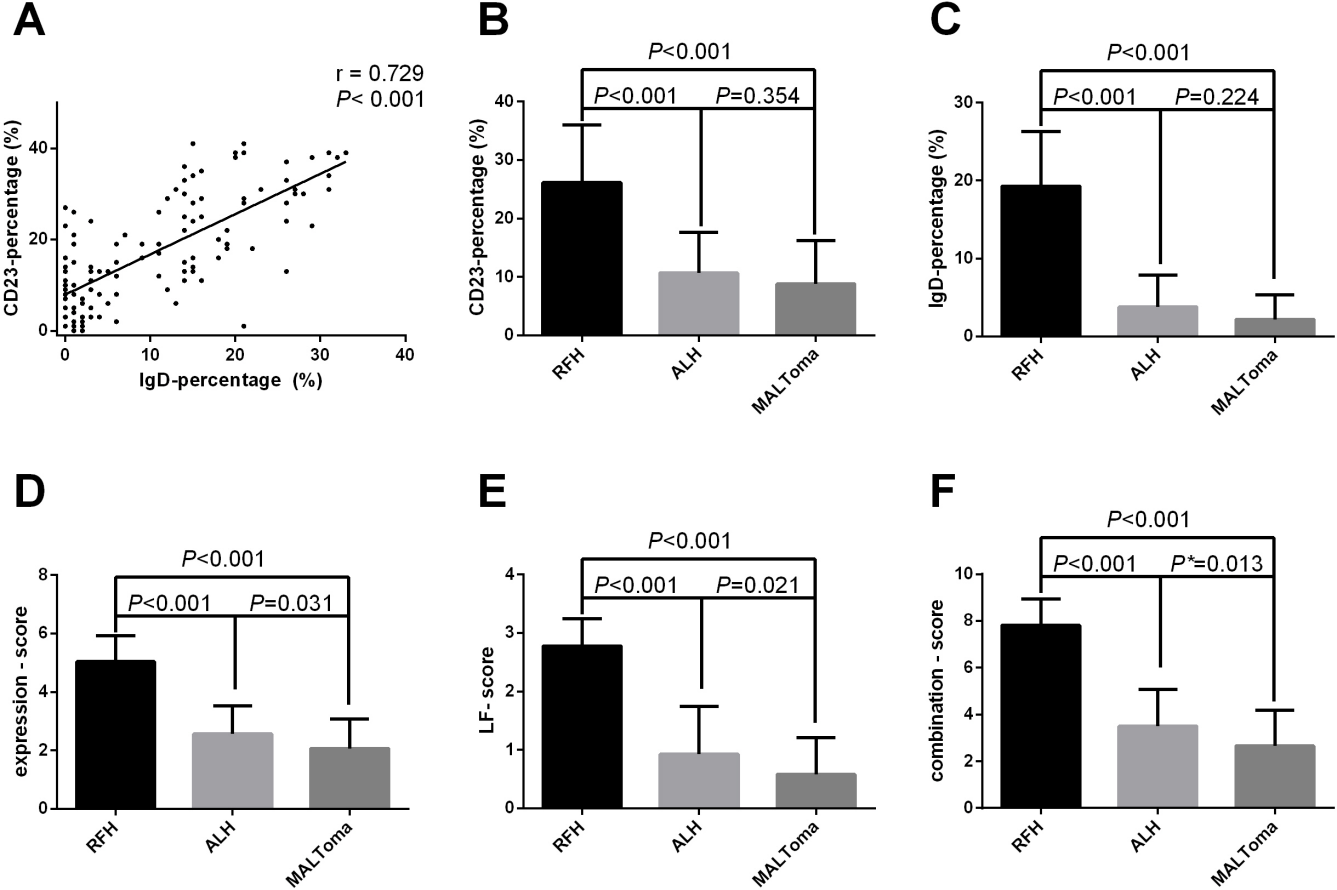
IgD and CD23 expression in RFH, ALH, and MALToma. (A) Pearson’s correlation between the IgD and CD23 staining rates was significantly positive among the three types of ocular adnexal LPDs (*r* = 0.729, *P* < 0.001). (B) The means and standard deviations of the CD23 positivity rates were evaluated and were shown in bar charts. The parameter was not statistical difference between ALH and MALToma. (C) The staining rate of IgD in RFH were much higher than those in ALH and MALToma (*P* < 0.01), whereas these parameters were not significantly different between ALH and MALToma. (D) The expression score of RFH were significantly higher than those of ALH or MALToma, (E) Similar differences among the three groups were found for the comparison of LF structure. (F) The difference in the combination score was significant between ALH and MALToma (*P* < 0.05/3). The asterisk (*) indicates a significant difference.

Based on the observed histomorphological differences described above, we first compared the expression of IgD and CD23 among the three types of LPDs. First, when considering all LPD cases, Pearson’s correlation was significant between the IgD and CD23 positive rates (Pearson’s *r* = 0.729; *P* < 0.001) (Fig 3A). In multiple comparisons, the rates of IgD and CD23 staining in RFH were significantly higher than those in the other two groups; however, there was no difference between ALH and MALToma in this respect (Fig 3B and Fig 3C). Then, based on the integrity of the LF structure and the expression levels of CD23/IgD, we individually established two scoring methods (LF score and expression score) for all samples according to the criteria shown in Tables 1 and 2. We found that both the expression score and LF score of RFH were significantly higher than those of ALH or MALToma, whereas no significant differences in IHC staining were observed between ALH and MALToma (Fig 3D and Fig 3E). Given that the integrity of the LF was confirmed by the IgD^+^/CD23^+^ expression profile of the cells and the structure of the LF, we combined the expression score and the LF score to establish a scoring method for LFs (combination score). The three types of LPDs were found to be effectively diagnosed using the combination score (*P* < 0.05 / 3) (Fig 3F).

**Fig 4:**
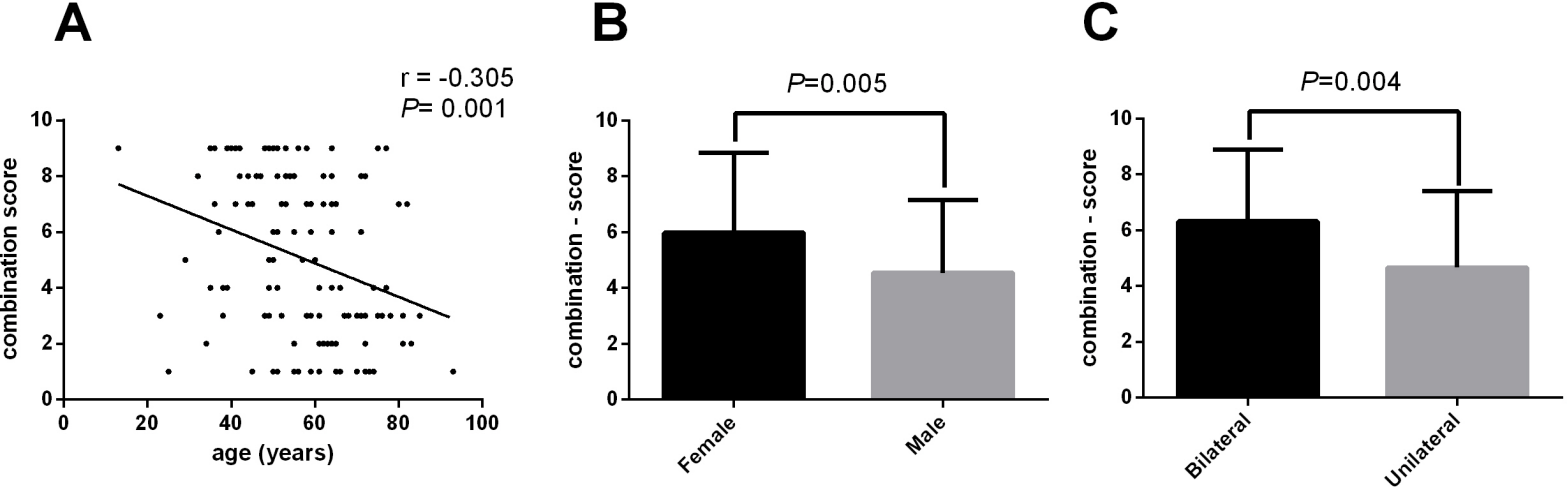
Combination score in LPDs and the correlation with clinical features. (A) The combination score decreased as the patient age increased, with a significantly negative correlation (*r* = −0.305; *P* = 0.001). (B) In all 125 cases with LPDs, the combination score of females was obviously higher than that of males (*P* = 0.005). (C) The combination score in patients with bilateral involvement was significantly higher than that in patients with unilateral involvement (*P* = 0.004).

In a further investigation, we analyzed the association between the combination score and the different clinical features described above. The combination score was negatively related with patient age (Pearson’s *r* = −0.305; *P* = 0.001) (Fig 4A). Additionally, we found that the combination score was higher in female patients than in male patients (Fig 4B). Furthermore, patients with bilateral involvement had a higher combination score than those with unilateral involvement (Fig 4C).

### IgH gene clonal rearrangement analysis

According to the BIOMED-2 protocol, we further detected IgH gene clonal rearrangements in 73 cases, including 35 RFH, 17 ALH, and 21 MALToma. Because some paraffin embedded samples had been stored for a long period, 52 of the DNA templates from the 125 cases were found to have become severely degraded by a quality control gene primer set test (S1 Fig). Therefore, the PCR amplification could not be carried out for these 52 cases. Monoclonal IgH gene rearrangements were detected in 18/21 MALToma specimens and 8 ALH specimens, but 9 ALH cases and all RFH lesions were negative for clonal rearrangements (S2 Fig). Based on a comparison with the results of this IgH gene clonal rearrangement analysis, the sensitivity of our combination scoring method was 85.7% (18/21) for MALToma samples and 35.3% (6/17) for ALH cases.

## Discussion

The differential diagnosis of the three types of LPDs poses a difficult clinical problem. In our study, we analyzed the clinical features of orbital LPDs and evaluated IgD/CD23 expression by IHC in a large sample. We further established a combination scoring method for the differential diagnosis of orbital LPDs.

Ocular adnexal LPDs are traditionally thought to be either benign reactive hyperplasias or malignant lymphomas. Because not all lymphoid lesions can be classified as benign or malignant, ALH is often described as the boundary between these two phenotypes. MALToma is the most frequent orbital malignancy in senior adults, with an increasing incidence according to several recent reports [2,4,28,29]. In the differential diagnosis of LPDs, it is often difficult to reach a definite diagnosis because of similarities in the clinical and histological features among ocular adnexal RFH, ALH, and MALToma, and especially between ALH and MALToma.

To our knowledge, this is the first report in which the clinical features of RFH, ALH, and MALToma of the ocular adnexa have been distinguished in such a large sample. MALToma patients are generally older [9,15], whereas RFH patients are often young or middle-aged. This suggests that younger individuals have a better controlled immune reaction to foreign antigens, which may help to prevent malignant transformation. Moreover, female patients comprise a major fraction of RFH cases, but only a small fraction of MALToma cases. This difference might be associated with differences in the immune system or hormonal variations between males and females, which would be an interesting topic for future studies. LPDs often involve two or more tissues of the ocular adnexa. In our study, RFH was more likely to be present in the bilateral orbit, especially in both lachrymal glands [16], whereas MALToma tended to be localized within the conjunctiva. A high incidence of conjunctival disease in MALToma cases was reported in a number of earlier studies [5,15,30–32]. The reduced lachrymal gland involvement in MALToma may be related to a reduced function of lachrymal glands in the elderly population. The pathophysiological basis for the increased involvement of the conjunctiva in MALToma is also unclear and should be addressed in future studies.

Diseases of particular ocular adnexal tissues often correspond to characteristic clinical presentations; however, because LPDs are relatively painless in the majority of cases, patients usually wait for a long time before seeking medical treatment. Because most elderly people are unable to identify small and painless alterations in their bodies, a yearly health examination is necessary to prevent high-grade transformations of MALToma.

In our clinical study, the differences in clinical features and progression were not obvious enough to distinguish the three disease types. Recently, several studies have suggested or provided supporting evidence for the concept that aberrant leukomonocyte proliferation may be subclinical until additional genetic changes cause the process to become irreversible [10, 33–38].

The ocular adnexa contains several mucosal surfaces of lymphoid tissue, such as the orbit, conjunctiva, lacrimal glands, and eyelids [4,15,39]. These regions are rich in antigenic stimuli, including infectious agents derived from the external environment; thus, the ocular region serves as a first line of defense against harmful antigens that enter the body and plays a crucial role in immunity. Consequently, it is common to find swollen conjunctiva, enlarged lacrimal glands, and/or orbital masses in these patients in the clinic. Orbital MALToma has typically been described in reactive and inflammatory conditions [40]. The treatment and prevention strategies for the majority of early MALTomas are not different from those for RFH (surgical excision and observation, respectively). Antibiotic therapy is often effective at shrinking the ocular lesion, which further suggests a potential relationship between the immune response to microbial pathogens and the genetic abnormalities leading to malignant transformation. From an etiological standpoint, it has been reported that some patients with ocular lesions have had one or more infectious diseases, although a definite causative relationship remains unclear [41]. The pathogens that may be relevant have been identified as *Chlamydia psittaci*, toxoplasma, Epstein–Barr virus, syphilis, human immunodeficiency virus, and hepatitis C [42–47]. The possible association between orbital LPDs and infectious agents is still heavily debated. The best example of an infection-associated LPD is *Helicobacter pylori* infection associated with gastritis and gastric MALToma [48–50], where chronic antigenic stimulation causes immunological responses and inflammation. However, not all lymphoproliferative conditions transition into malignant lesions, suggesting that this is a complex process dependent on various factors, including the patient’s age, immune system response, exposure duration, and stimulus length.

LFs are crucial for the generation of humoral immunity [17,18]. However, although LFs provide B-cells for intact antibody-dependent responses, they are associated with a risk of generating autoreactive B-cells and B-cell lymphomas. Various lymphomas have been hypothesized to arise from these cells based on their differentiation stages within the LFs [51]. For instance, hyperplasia of mature B-cells that localize to the marginal and interfollicular zones likely results in MALToma through mutations incurred by immunoglobulin class-switch recombination [52,53]. This mechanism could explain the low-grade feature of MALToma. Conversely, GC B-cells within the FDC network are characterized by a high rate of proliferation, whereas GC cell hyperplasia is restricted by the FDC network [54,55]. Because most naïve mature B-cells in the MZ express IgD, and given that FDC networks have typical CD23 expression patterns, the combined use of IgD and CD23 immunostaining can clearly reveal the LF structure.

In our IHC study, our combination scoring system was useful to assess the alterations in the amorphous LF structure to better distinguish among RFH, ALH, and MALToma. In RFH, the profiles of CD23^+^ GCs and IgD^+^ MZs were distinct and easily recognizable. In ALH, the number and shape of the LFs were respectively decreased and varied, with irregularities in the size and presence of IgD^+^ mantle circles and CD23^+^ FDC networks. LFs were also found in MALToma, but with a significantly reduced incidence. In addition, only scattered and small residual CD23^+^ FDC networks and lamellar IgD^+^ cell clusters were observed in MALToma. This intriguing phenomenon of changes in the characteristics of LFs among the three groups also suggests that the LF structure becomes gradually disrupted as the disease state progresses. The results of this retrospective study are consistent with those of some previous studies [5,30,56].

IgH gene rearrangement analysis is sensitive and helpful for evaluating some patients for whom it is difficult to make a final diagnosis, especially regarding the stage of ALH. Some studies have shown that novel IgH gene rearrangements of several ALH cases could be detected in further follow-up biopsies [20,57]. The combined detection of three gene rearrangements (IgH-FRl, -FR2, and -FR3) is helpful for making a final diagnosis. However, the analysis of complex IgH gene rearrangements is expensive and difficult to apply in some primary hospitals. Our scoring method, which is based on morphological evidence, might be easier to perform and may assist with the diagnosis of MALToma as well as the differential diagnosis of LPDs. In summary, from an analysis of 125 LPD cases, we found that IHC staining for IgD and CD23 is a useful tool for distinguishing among RFH, ALH, and MALToma. The diagnostic scoring method that we designed could be used to classify most ocular adnexal B-cell clonal proliferative diseases. Ultimately, a combination of clinical features and histologic evaluation will provide the best opportunity to correctly diagnose LPDs and determine an appropriate treatment strategy. Nevertheless, additional studies are necessary to verify our findings and conclusively determine the pathogenetic relationship among the various B-cell LPD subtypes.

## Supporting information

S1 Fig Quality control of DNA samples. Lane 6: DNA marker; Lanes 5, 8, 9, 11–14, and 18: clear 200, 300, 400, and 600 bp PCR product bands, indicating that the template DNA was extracted successfully; Lanes 1–4, 7, 10, and 15–17: no specific bands or thin bands, indicating that the amount and/or quality of the extracted DNA was insufficient for analysis.
(JPG)

S2 Fig Polyacrylamide gel electrophoresis to detect IgH gene rearrangements in ocular adnexal LPDs. **(**A) IgH-tube A. Lane 1: DNA marker, Lane 2: positive control, Lane 3: negative control, Lane 4: blank control. Lanes 5–6 show amplified bands between 310–360 bp, indicating a positive IgH-tube A gene rearrangement. Lanes 7 and 8 show no clonal rearrangement. (B) IgH-tube B. Lane 1: DNA marker, Lane 9: positive control, Lane 10: negative control, Lane 11: blank control. Lanes 12–14 show amplified bands between 250–295 bp, indicating a positive IgH-tube B gene rearrangement. Lane 15 shows no clonal rearrangement. Lane 16 shows smear bands, indicating a polyclonal rearrangement.
(C) IgH-tube C. Lane 1: DNA marker, Lane 17: positive control, Lane 18: negative control, Lane 19: blank control. Lanes 20–21 show amplified bands between 100–170 bp, indicating a positive IgH-tube C gene rearrangement. Lanes 22–24 show no clonal rearrangement.
(TIF)

S1 File File contains full data for each subject included in the study. (XLSX)
