# Supplementary figures and images for: Establishment of a combination scoring method for diagnosis of ocular adnexal lymphoproliferative disease
Source: PLoS One. 2017 May 16;12(5):e0160175. doi: 10.1371/journal.pone.0160175 (PMC5433690; doi:10.1371/journal.pone.0160175)

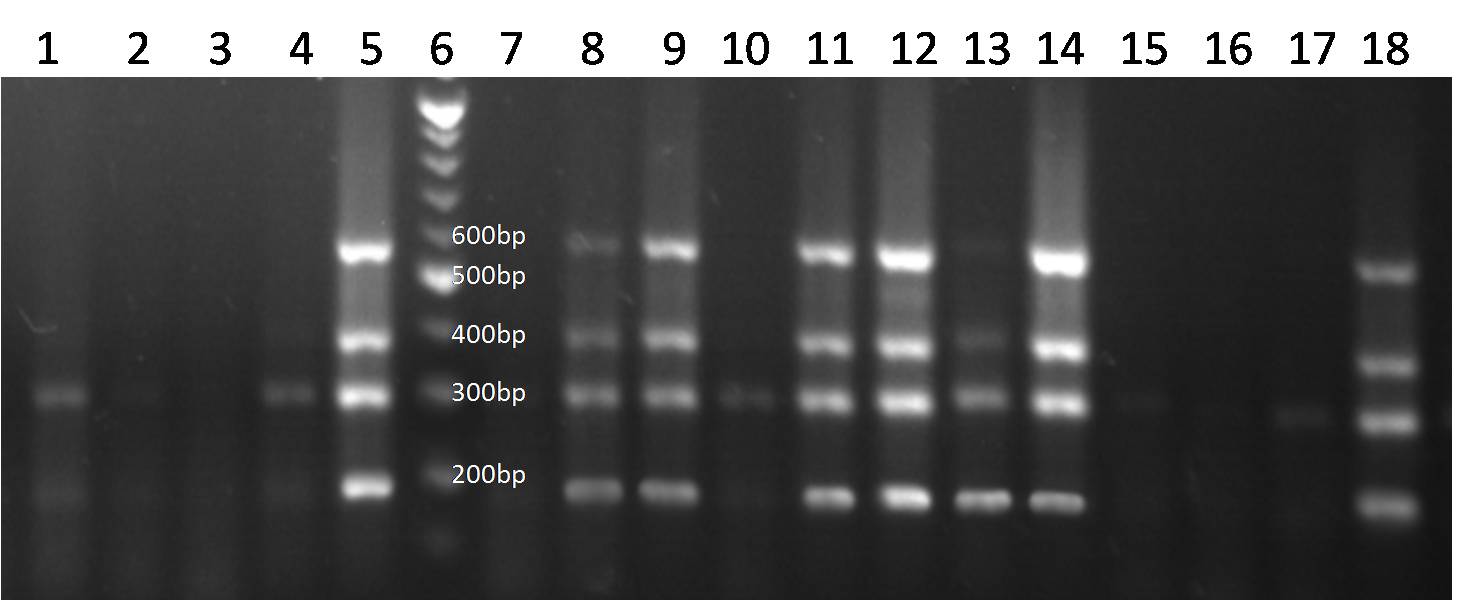

Supplement: S1 Fig — Lane 6: DNA marker; Lanes 5, 8, 9, 11–14, and 18: clear 200, 300, 400, and 600 bp PCR product bands, indicating that the template DNA was extracted successfully; Lanes 1–4, 7, 10, and 15–17: no specific bands or thin bands, indicating that the amount and/or quality of the extracted DNA was insufficient for analysis. (JPG) [file pone.0160175.s001.jpg]

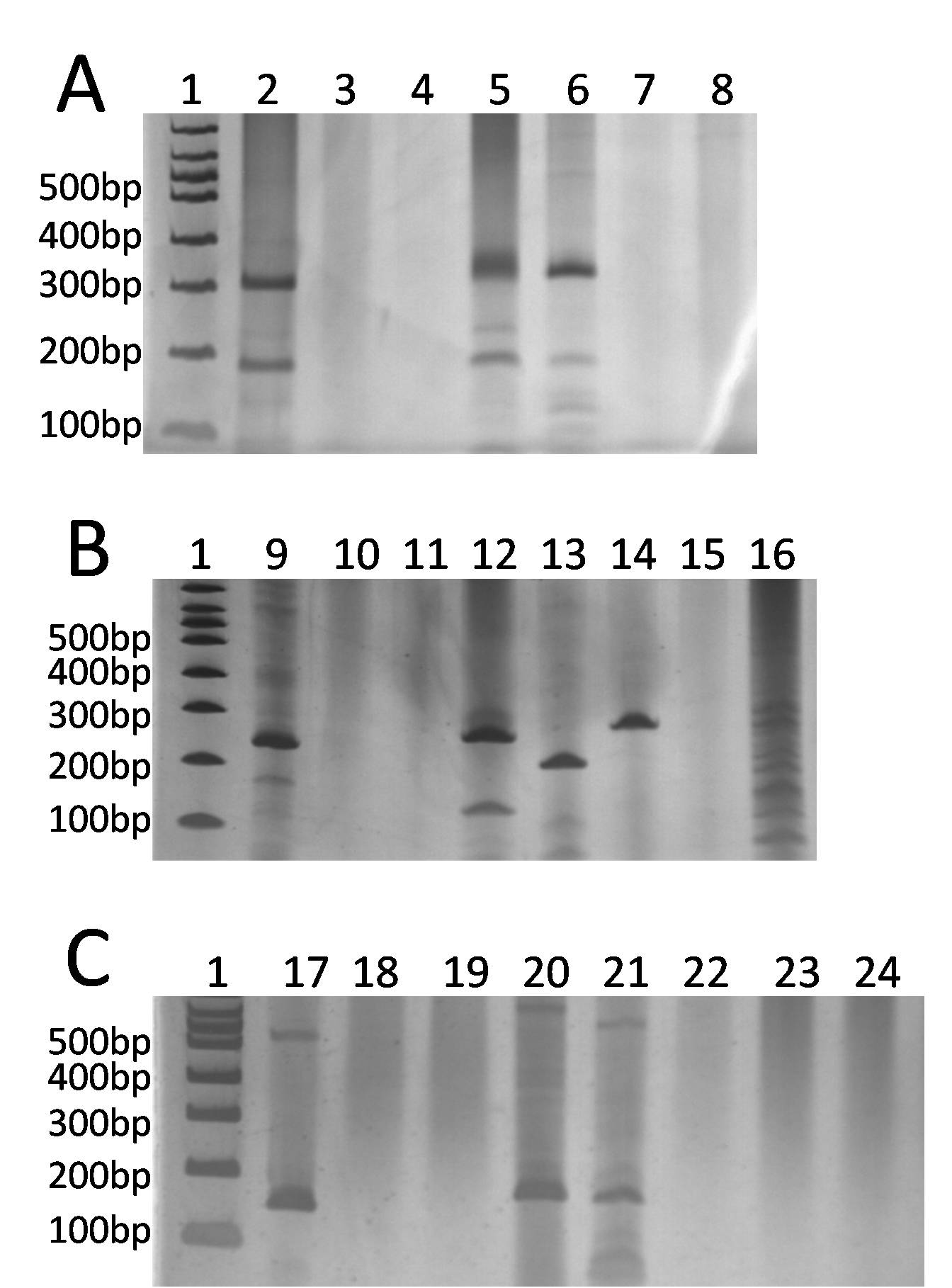

Supplement: S2 Fig — (A) IgH-tube A. Lane 1: DNA marker, Lane 2: positive control, Lane 3: negative control, Lane 4: blank control. Lanes 5–6 show amplified bands between 310–360 bp, indicating a positive IgH-tube A gene rearrangement. Lanes 7 and 8 show no clonal rearrangement. (B) IgH-tube B. Lane 1: DNA marker, Lane 9: positive control, Lane 10: negative control, Lane 11: blank control. Lanes 12–14 show amplified bands between 250–295 bp, indicating a positive IgH-tube B gene rearrangement. Lane 15 shows no clonal rearrangement. Lane 16 shows smear bands, indicating a polyclonal rearrangement. (C) IgH-tube C. Lane 1: DNA marker, Lane 17: positive control, Lane 18: negative control, Lane 19: blank control. Lanes 20–21 show amplified bands between 100–170 bp, indicating a positive IgH-tube C gene rearrangement. Lanes 22–24 show no clonal rearrangement. (TIF) [file pone.0160175.s002.tif]
